# AI-Driven Risk Prediction Tool (TSP-9) Informs Risk-Aligned Care for Patients with Barrett’s Esophagus

**DOI:** 10.3390/diagnostics15212776

**Published:** 2025-10-31

**Authors:** Jay N. Yepuri

**Affiliations:** Digestive Health Associates of Texas, Bedford Central Drive, Bedford, TX 76022, USA; jay.yepuri@gmail.com

**Keywords:** artificial intelligence, Barrett’s esophagus, non-dysplastic Barrett’s esophagus, risk stratification, esophageal adenocarcinoma

## Abstract

**Background and Clinical Significance**: Barrett’s esophagus (BE) is the precursor to esophageal adenocarcinoma (EAC). Accurately predicting which patients with BE are at the highest risk of progressing to EAC is a significant clinical challenge. This article discusses how the tissue systems pathology test (TSP-9, TissueCypher) can help guide risk-aligned care for patients with BE. TSP-9 is an AI-driven prognostic test that stratifies patients with BE for risk of progression to high-grade dysplasia (HGD)/EAC. **Case Report Presentation**: Three clinically low-risk patients had esophageal biopsies tested by TSP-9. The real-world utility of TSP-9 is demonstrated through a brief discussion of how the test was utilized to assess each patient’s personalized risk of BE progression to HGD/EAC and inform risk-aligned care. **Conclusions**: The use of validated AI-powered tools such as TSP-9 is poised to become standard practice in gastroenterology clinical settings and will help improve health outcomes for patients with BE to prevent EAC-related mortality.

## 1. Introduction

Advances in computing and data science have enabled rapid development and integration of artificial intelligence (AI) into many areas of medicine, including analysis of medical images. As a result, vast amounts of objective and quantitative information can be extracted from medical images, and the management of diseases traditionally reliant on subjective and qualitative interpretation of visual cues for diagnostic and prognostic purposes are poised to be transformed. Barrett’s esophagus (BE) is one such disease. Gastroenterologists and pathologists alike have long noted the need for better prognostic approaches to managing their patients with BE [1,2] as evidenced through a brief literature review. The management approach applied to the case series herein employs a validated prognostic spatialomics test to inform patient care. The test evaluates proteomics and morphology captured by digital imaging and analyzes the data using AI to provide clinically actionable information that informs clinical management decisions and addresses the limitations of traditional BE management.

### 1.1. Review of Traditional BE Management

BE is a premalignant condition that is the only known precursor to esophageal adenocarcinoma (EAC), which has a poor prognosis of 80% mortality within 5 years of diagnosis [3]. BE is caused by chronic exposure of the esophagus to stomach acid associated with gastroesophageal reflux disease (GERD), which causes the stratified squamous epithelium that normally lines the esophagus to be replaced by columnar-lined epithelium resembling that of the small intestine. Current clinical society guidelines [4,5,6] recommend individuals with risk factors for development of BE including GERD, age > 50 years, being non-Hispanic white, a history of smoking, obesity, being of the male sex, and a family history of BE or EAC [7,8] be screened by esophagogastroduodenoscopy (EGD). If signs of BE are present, pinch biopsies are obtained and microscopically reviewed by a pathologist. BE is diagnosed if the presence of intestinal metaplasia (IM) is observed within the tubular esophagus [9]. BE is pathologically graded based on the presence and level of neoplasia, including non-dysplastic (ND) BE, low-grade dysplasia (LGD), high-grade dysplasia (HGD), intra-mucosal cancer (IMC), and invasive EAC. BE can also be assessed as indefinite for dysplasia (IND) when significant inflammation precludes a definitive diagnosis upon pathological review [9,10]. Limitations to pathological diagnoses of BE include significant interobserver variability in the visual interpretation and grading of dysplasia in BE [6,11], prompting the recommendation for expert confirmation in ambiguous or dysplastic cases [6]. These issues with concordance are well-documented amongst both general and expert gastroenterological (GI) pathologists [11,12,13] and demonstrated by the diagnostic downstaging that often occurs in cases of LGD [12,14].

A confounding issue in managing patients diagnosed with BE is predicting which patients will progress to HGD/EAC, and which will not. Current clinical guidelines use population-based risk assessments to provide guidance on clinical management strategies to detect progression early, recommending patients diagnosed with BE undergo routine endoscopic surveillance with biopsies at varying intervals (every 6 months to every 5 years) depending on the patient’s grade of BE diagnosis [4,5,6] and their clinicopathological risk factors, including BE segment length [6]. Using this traditional population-based approach, each escalating grade of BE is associated with a patient having an increased 5-year risk of progressing to HGD/EAC, from approximately 3.2% for those with NDBE, 7.5% for those with IND, to 8.7% for those with LGD [15,16,17]. Patients diagnosed with HGD have the highest risk of progression to EAC, reported to be 33% over 5 years [18]. Due to the high risk of developing EAC, patients definitively diagnosed with LGD or HGD are recommended to have their BE tissue removed by endoscopic eradication therapy (EET), rather than continued management of their BE through routine surveillance alone [4,5,6]. This population-based approach to risk assessment is widely recognized to be ineffective, with the majority of patients with BE not undergoing EET and, as such, there has been only modest success in reducing EAC-related mortality in recent decades [3,19]. Population-based risk estimates may underestimate the progression risk for subsets of women, younger individuals, and those without a history of GERD [20,21,22], as well as patients with NDBE who represent most of the individuals living with BE. Individually, there are patients with a diagnosis of NDBE, who are typically deemed low-risk, but have a high individual risk of progression to HGD/EAC and would benefit from EET [19] if they were identified as high-risk using available prognostic testing.

### 1.2. Leveraging AI for Risk Stratifying Patients with BE

The tissue systems pathology test (TSP-9; TissueCypher) is an AI-powered test that was created to improve risk stratification in patients with BE and provide clinicians with a personalized, rather than a population-based, approach to objectively predict which of their patients with BE are at a high risk of progressing to HGD/EAC within 5 years [23,24,25,26,27,28,29,30].

**Figure 1:**
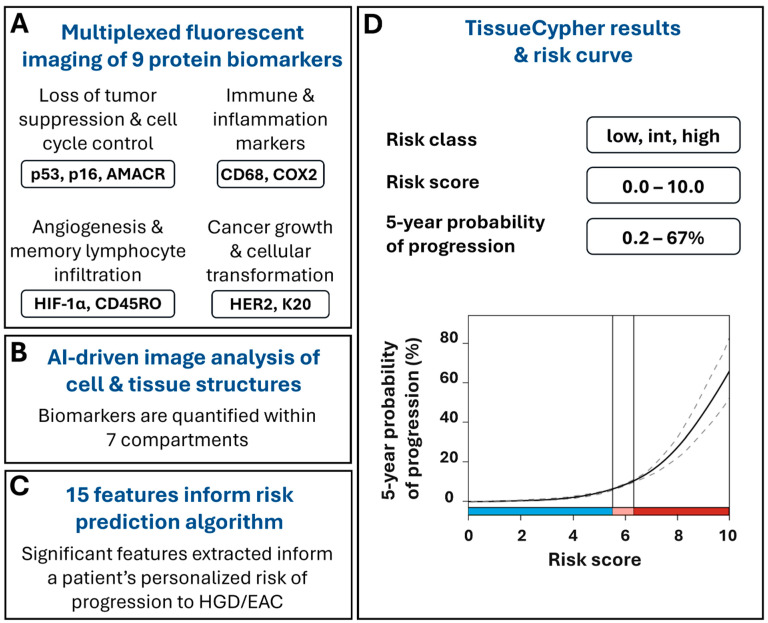
TSP-9 (TissueCypher) utilizes quantitative immunofluorescence and AI-driven spatialomics to predict a patient’s personalized 5-year risk of progression to HGD/EAC. (**A**) Biopsies obtained from EGD that have received a pathological diagnosis of NDBE, IND, or LGD are eligible for TSP-9 testing. FFPE blocks or unstained slides are sent to an accredited, centralized laboratory where biopsy slides are immunofluorescently labeled for 9 protein biomarkers involved in cancer growth and cellular transformation, the immune response and inflammation, angiogenesis and memory lymphocyte infiltration, and cell cycle control and tumor suppression and imaged by whole slide fluorescence scanning. (**B**) Biomarkers are automatically quantified within nuclei, cytoplasm, cells, epithelial nuclei, epithelium, stroma, and metaplastic epithelium and (**C**) the resulting data are integrated into 15 spatialomics features that inform the risk-prediction algorithm. (**D**) Clinicians are provided with a patient’s personalized risk score, risk class, and 5-year probability of progression to HGD/EAC. Scores corresponding to low-risk (blue), intermediate-risk (pink), and high-risk (red) classes are visually represented along the x-axis of the risk curve.

The test uses formalin-fixed, paraffin-embedded (FFPE) BE tissues obtained during screening and surveillance endoscopies to detect and quantify multiple changes that can occur at the molecular and cellular level as BE progresses to EAC, prior to the appearance of morphological changes that can be detected by a pathologist. TSP-9 is a laboratory-developed test performed in a Clinical Laboratory Improvement Amendments (CLIA)-certified, College of American Pathologists (CAP)-accredited, New York State Department of Health-approved laboratory (Pittsburgh, PA, USA). The FFPE tissues undergo automated multiplexed nuclear and immunofluorescent labeling of nine protein biomarkers that are involved in cell cycle control and tumor suppression (p53, p16, AMACR), neoplastic growth and cell transformation (HER-2, CK20), as well as inflammation, immune system regulation, and angiogenesis (CD45RO, CD68, COX2, HIF-1α) [31] (Figure 1A). The slides undergo whole slide imaging and automated image analysis, including the use of Densely Connected Neural Networks (DenseNet), to identify intact tissue areas containing BE mucosa, excluding non-BE tissue and artifacts that may arise during specimen processing, and computer vision algorithms to identify and segment 7 cell and tissue structures within the digital images, including cells, cytoplasm, nuclei, epithelium, epithelial nuclei, metaplastic epithelium, and stroma (Figure 1B) [31,32,33,34]. The custom image analysis software pipeline then automatically extracts and analyzes high-dimensional quantitative information from 15 spatialomics features indicative of early molecular and cellular signatures of progression to HGD/EAC [23]. The quantitative information is subsequently integrated into a validated and locked risk-prediction algorithm (Figure 1C) to report a patient’s personalized risk score (0–10), risk class (low, intermediate, high), and the probability of having their BE progress to HGD/EAC within 5 years (Figure 1D) [23,24,25,26,27,28,29,30].

### 1.3. Model Description, Training, and Validation

The development and validation of the risk-prediction model used in the TSP-9 platform was previously described [23,31]. Briefly, FFPE biopsy samples were obtained from patients with BE (diagnoses of NDBE, IND, and LGD) who exhibited incident progression to HGD or EAC (progressors), or who did not progress to HGD/EAC (non-progressors). Samples were attained from multiple institutions with broad geographic distribution with samples from Europe and the U.S. comprising 55% and 45% of the total, respectively. Patient age, sex, and race were characteristic of the general BE population [23]. The low progression rate of BE to HGD/EAC necessitated the use of a retrospective case–control study design.

Samples were randomly assigned to the training or validation set to mitigate selection bias. Performance of multiplexed nuclear and immunofluorescent labeling was conducted on FFPE sections followed by whole slide image analysis. Proprietary algorithms segmented cell- and tissue-based objects and quantified the expression and spatial distribution of biomarkers within areas of interest to produce 13,538 features per biopsy [23,31]. Univariate conditional logistic regression (CLR) was performed on the training set of imaging data from 41 progressors and 142 non-progressors to compare BE progressors to non-progressors (control) to enable feature selection for multivariable model building, and features were combined into classifiers. Seventeen features were chosen for further use based on *p* values from the univariate CLR comparisons of cases vs. controls. Leave-one-out cross validation (LOOCV) was performed, and the prediction model was developed in the training set by the sum of the features weighted by the univariate Cox coefficients to prevent overfitting. LOOCV resulted in the identification of a model producing the highest concordance-index which utilized 15 features, and the definition of a risk score for each patient ranging from 0.0 to 10.0. Cutoffs were determined to stratify patients into low-, intermediate- and high-risk classes [23]. All parameters of the test were ‘locked’ after the training set analysis was complete. The resulting model was validated using a quarantined independent validation set of samples from 38 progressors and 145 non-progressor controls. High prognostic accuracy of the test is indicated by an area under the receiver operating characteristic curve (AUROC) of 0.87 for the classifier [23]. TSP-9 has since been clinically validated in multiple independent, international, and multi-center studies [23,24,25,26,27,28,29,30]. Overall performance metrics for the test are derived from a pooled analysis of data from 699 unique patients. The test has a sensitivity of 62.3%, a specificity of 79.8%, a prevalence-adjusted negative predictive value (NPV_adj_) of 97.4%, and a prevalence-adjusted positive predictive value (PPV_adj_) of 25.1% [26]. The clinical utility of the test in patient management has also been previously described [35,36,37,38].

## 2. Case Report Presentation

**Figure 2:**
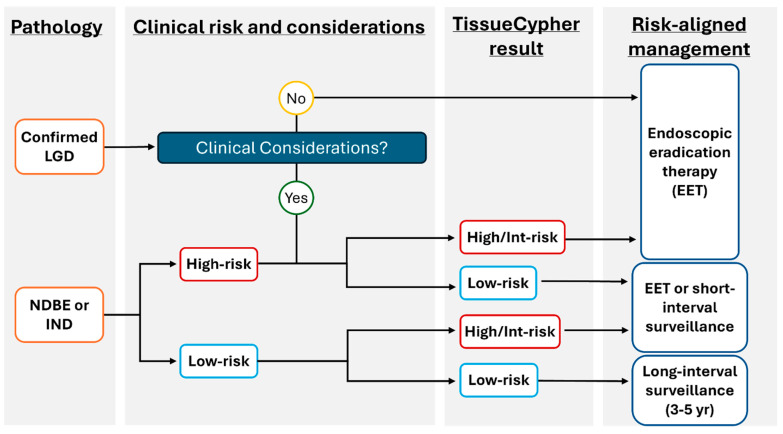
Proposed model to incorporate TSP-9 (TissueCypher) into clinical practice to inform risk-aligned decision making for patients with BE. Risk-aligned management in BE can be highly personalized to improve patient health outcomes. After initial stratification of patients by pathological diagnosis, clinical considerations that may influence patient management (including age/co-morbidities that may affect a patient’s ability to undergo serial sedation for surveillance or EET) are weighed. The prognostic information provided by TissueCypher informs physician–patient discussions to support risk-aligned care for patients with BE.

The test incorporating AI for risk stratification of patients with BE is now readily accessible and can be implemented into a gastroenterologist or pathologist’s clinical practice through the TSP-9 test (Figure 2). The use of this personalized medical test addresses many insufficiencies with current traditional BE management without objective risk stratification tools, as reviewed above. The following case series highlights how personalized risk stratification using TSP-9 informed the clinical management plans for three patients with BE.

### 2.1. Case 1: Low-Risk TSP-9 Score Supported De-Escalation of Care for a Patient with NDBE

A 19-year-old female with no BE-relevant medical history aside from nocturnal GERD and intermittent dysphagia was seen at a gastroenterology center. An index EGD revealed mild esophagitis (LA Grade A) in the distal esophagus and a BE segment of C0M1 (Prague criteria: circumferential BE: 0 cm; maximum extent BE: 1 cm). Biopsies evaluated from the segment returned a pathology diagnosis of NDBE. Clinical risk factors such as the patient’s age, sex, and short BE segment length would classify this patient as low-risk for progression to EAC [4,5,6]. Due to the patient’s young age, her family was heavily involved in this patient’s care, and the resulting diagnosis was worrisome for both the patient and her family. This prompted additional conversations regarding the most appropriate management strategy to mitigate concern. Traditional society guidelines recommend a 5-year surveillance interval for patients with short segment NDBE [4,5,6], but concern regarding the patient’s symptoms, presence of BE at such a young age, and increased anxiety led to the suggestion of yearly surveillance as the management strategy for this patient. In addition to extensive physician–patient discussions, the TSP-9 test was ordered to provide additional information to help guide the management of this patient.

FFPE blocks of the esophageal pinch biopsies were sent for evaluation by TSP-9, which returned a risk score of 4.1, corresponding to a low-risk class and a 5-year probability of progression of 3% for this patient (Table 1). Receipt of the low-risk TSP-9 result reduced BE-related anxiety for both the patient and her family. The objective result also inspired confidence in de-escalating care for this patient, reducing surveillance from a yearly interval to a 3-year surveillance schedule to manage her BE. In this case, the TSP-9 test provided unambiguous evidence that increased physician and patient confidence regarding de-escalation of care using a personalized surveillance interval.

### 2.2. Case 2: High-Risk TSP-9 Score Informed an Escalation of Management for a Patient with NDBE

A 75-year-old female patient with a history of cancer that was in remission and several unrelated co-morbidities presented with chronic GERD controlled by PPIs (40 mg/daily). A previous EGD revealed esophagitis (LA Grade A) and a biopsy from a C1M1 segment was diagnosed as NDBE. Traditional guideline-based management would consider this patient low risk, informing a 5-year surveillance interval. At follow-up EGD, the patient’s BE segment advanced to 6 cm and a hiatal hernia was noted. Biopsies from the elongated segment were also diagnosed as NDBE upon pathology review. As this patient was still considered clinically low-risk due to clinical factors and a lack of dysplasia, a 3-year surveillance interval was recommended by the patient’s previous GI care team.

Complicating this case was the intermittent, non-contiguous nature of this patient’s GI care. The patient had fallen out of routine management with a GI physician; the patient’s primary care physician helped to reestablish an appropriate management plan. An additional, subsequent EGD performed revealed a drastic lengthening of the BE segment (C10M10) that was again diagnosed as NDBE upon review. Traditional, guideline-based management would be a follow-up EGD in 3 years [4,5,6]. Due to this patient’s intermittent care and lengthening of the BE segment without dysplastic progression, it was decided that adjunctive information would be useful to best manage this patient. Esophageal biopsies from the C10M10 segment were sent for TSP-9 testing, which returned a high-risk score of 7.1, corresponding to a 17% probability of progression within 5 years (Table 1).

This high-risk score informed additional, in-depth physician–patient discussions regarding the patient’s BE, individualized risk of progression, and clinical factors that might complicate BE treatment, such as advanced age. Ensuring that the patient is medically fit to undergo EET is of utmost importance, as in long-segment BE cases, multiple rounds of ablative therapy are typically necessary to effectively eradicate the BE segment. Therefore, it is vital to ensure that the risks of serial therapeutic endoscopies with sedation do not outweigh the benefits of ablating the BE tissue due to risk of progression. The information provided by the high-risk TSP-9 score helped to support escalation of care to EET in this patient rather than management by surveillance alone. EET was initiated as a safe and effective method for eradicating BE tissue in this patient with the goal of preventing EAC [39,40,41].

### 2.3. Case 3: TSP-9 Low-Risk Score Informed Risk-Aligned Management in a Patient Diagnosed with NDBE

A 52-year-old male patient with a history of chronic, well-managed GERD (40 mg/daily PPI) presented for care at a GI center with significant anxiety regarding a BE diagnosis. This patient was concerned about the potential of long-segment BE and progression to EAC, and he preemptively wished to be considered as a candidate for EET to alleviate these concerns. Upon intake, the patient reported to be a former smoker and had a documented history of alcoholic cirrhosis, which was complicated by portal hypertension. This raised additional concern regarding the patient’s insistence on ablative therapies to manage BE, as his cirrhosis would elevate his risk for complications from the serial procedures required for EET.

The patient underwent an EGD that revealed a BE segment of C2M5. Biopsies from this segment were diagnosed as NDBE upon pathological review. Based on this patient’s clinical profile and diagnosis of NDBE, a 3–5 year surveillance interval was considered in alignment with society guidelines [4,5,6]. However, due to the patient’s anxiety and preference for EET, this was considered in the initial management strategy, and adjunctive information for risk of progression was sought by ordering the TSP-9 test. Biopsies from the patient’s BE segment were sent for TSP-9 testing, which returned a low-risk score of 4.1, corresponding to a 3% probability of progression to HGD/EAC in 5 years (Table 1). The TSP-9 test results enabled a risk–benefit discussion between the patient and physician and led to the clinical decision that a less aggressive management strategy could be safely pursued. The patient’s anxiety regarding progression was alleviated and the TSP-9 result inspired confidence in the management of BE through a personalized surveillance interval. Ultimately, a 3-year long-term surveillance interval was selected to provide risk-aligned management in lieu of EET for this patient.

## 3. Discussion

The traditional approach to risk stratifying patients with BE is based on histopathological grading of patient BE samples and patient clinicopathological factors. There are significant limitations to this approach, including interobserver variability in histopathological grading and the inability of histopathology to accurately determine which patients with NDBE are at high or low risk of progressing to HGD/EAC. The approach is also reliant on population-based rates of progression to HGD/EAC that may not apply to all individuals with BE, including those who are female, non-white, non-obese, without a history of GERD, and <50 years of age.

TSP-9 is an extensively validated test that quantifies early molecular and cellular signatures to objectively predict the individualized risk of patients with BE progressing to HGD/EAC within 5 years. The performance of TSP-9 is independent of clinicopathological factors and has been demonstrated to be the strongest predictor of progression to HGD/EAC for patients with BE [23,26,29,30]. The TSP-9 test identifies patients with BE at a biologically low-risk of progression to HGD/EAC which enables clinicians to de-escalate care to reduce unnecessary surveillance procedures and alleviate disease-associated anxiety. TSP-9 also identifies patients diagnosed with NDBE considered low-risk on a population basis for progression to have a biologically high risk of progressing to HGD/EAC to guide escalation of care to EET with the goal of reducing EAC-related mortality [23,24,25,26,29,35,36,42]. Ongoing National Institutes of Health (NIH)-funded, multi-center studies including SURVENT (Surveillance vs. Endoscopic Therapy for Barrett’s Esophagus With Low-grade Dysplasia) and SCRiBE (Stratification of Cancer Risk in Patients with Non-Dysplastic Barrett’s Esophagus using TissueCypher) are being conducted to further independently validate the performance and reproducibility of the proprietary TSP-9 risk-prediction algorithm [43].

TSP-9 was the first AI-driven risk stratification test of its kind for patients with BE but is now joined by other tests and technologies incorporating AI to improve patient care. Additional uses of AI in gastroenterology include Esopredict, a molecular-based approach to risk-stratifying patients with BE that also utilizes an AI-driven risk-prediction algorithm [44,45,46]. Development of the test speaks to the growing adoption of AI in BE management, and published studies describing this methylation-based assay [44,45,46] are promising for its future clinical applications. Endoscopists have also used AI to aid in identifying areas of concern in the esophagus, encouraging thorough examination and sampling to promote early detection of dysplasia and prevent progression to EAC [47,48,49,50]. Multiple academic research groups with streamlined access to electronic health records have likewise investigated AI’s capacity to collect, integrate, and analyze large data sets to identify patients with BE who are at risk of progressing to EAC and help automate clinical decision making regarding endoscopic surveillance timing [51,52]. AI is also being developed to improve the histopathological diagnosis of BE [53,54,55]. Similar applications of AI are being explored in other areas of medicine [56,57], and increased use of these tools in different clinical specialties stands to improve public health through rapid disease detection, accurate diagnoses, and personalized treatment plans to improve patient health outcomes.

## 4. Conclusions

The three real-world cases outlined herein demonstrate how valuable, adjunctive information provided by an AI-driven prognostic test such as TSP-9 can improve clinical management of patients with BE by informing physician–patient discussions and supporting risk-aligned care. The use of AI-driven tools such as TSP-9 is transforming how gastroenterologists and pathologists provide risk-aligned care to their patients. The adoption of similar prognostic tools is well-positioned to provide invaluable clinical utility for patients in other disease states in the years to come.

## Figures and Tables

**Table 1:** Patient demographics and clinical management.

	Case 1	Case 2	Case 3
Age (years)	19	75	52
Sex	Female	Female	Male
Ethnicity	Caucasian	Caucasian	Caucasian
Relevant family history	No	No	No
Smoker	No	No	Former
GERD	Nocturnal	Chronic	Chronic
PPIs	No	40 mg/daily	40 mg/daily
Reported co-morbidities	None	Well-controlled BPD, hyperlipidemia; anal squamous cell cancer in remission	Well-controlled GERD, alcoholic cirrhosis
Reason for initial visit	Intermittent solid dysphagia; reflux	Unknown	BE-related anxiety
1st EGD	C0M1/NDBE	C1M1/NDBE	C2M5/NDBE
2nd EGD	N/A	C1M6/NDBE	N/A
3rd EGD	N/A	C10M10/NDBE	N/A
Clinical Profile	Low-risk	Low-risk	Low-risk
Initial Proposed Management	1-year surveillance interval	5-year surveillance interval	3-year surveillance interval
TissueCypher Risk Class	Low-risk	High-risk	Low-risk
TissueCypher Risk Score	4.1	7.1	4.1
TissueCypher 5-year Probability of Progression	3%	17%	3%
TissueCypher-informed Management	3-year surveillance	EET	3-year surveillance

Abbreviations: bipolar disorder (BPD), endoscopic eradication therapy (EET), esophagogastroduodenoscopy (EGD), gastroesophageal reflux disease (GERD), not applicable (N/A), non-dysplastic Barrett’s esophagus (NDBE), proton pump inhibitor (PPI).

## Data Availability

Data generated for this article (identifiable health data) is not publicly available due to privacy, legal, and ethical reasons. The original contributions presented in this study are included in the article. Further inquiries can be directed to the corresponding author.
